# Copy-number-gain of telomerase reverse transcriptase (hTERT) is associated with an unfavorable prognosis in esophageal adenocarcinoma

**DOI:** 10.1038/s41598-023-44844-7

**Published:** 2023-10-17

**Authors:** Su Ir Lyu, Felix C. Popp, Adrian Georg Simon, Anne Maria Schultheis, Thomas Zander, Caroline Fretter, Wolfgang Schröder, Christiane J. Bruns, Thomas Schmidt, Alexander Quaas, Karl Knipper

**Affiliations:** 1 Faculty of Medicine and University Hospital of Cologne, Institute of Pathology, University of Cologne, Cologne, Germany; 2 Faculty of Medicine and University Hospital of Cologne, Department of General, Visceral and Cancer Surgery, University of Cologne, Cologne, Germany; 3 Faculty of Medicine and University Hospital of Cologne, Department of Internal Medicine, Center for Integrated Oncology Aachen-Bonn-Cologne-Duesseldorf, University of Cologne, Cologne, Germany

**Keywords:** Oesophageal cancer, Tumour biomarkers

## Abstract

Esophageal adenocarcinoma exhibits one of the highest mortality rates among all cancer entities. Multimodal therapy strategies have improved patients’ survival significantly. However, patients in early stages are currently limited to receiving only local therapies, even though some patients within this group showcase short survival periods. Until now, there has been no widely established clinically used biomarker to detect these high-risk patients. Telomerase reverse transcriptase (*TERT*), a gene encoding a crucial subunit of the telomerase enzyme, plays a significant role in establishing cancer cell immortality and is under suspicion for its potential contribution to tumor progression. Therefore, we aimed to evaluate the clinical relevance of the *TERT* amplification status. We included 643 patients with esophageal adenocarcinoma, who underwent Ivor-Lewis esophagectomy at the University Hospital of Cologne. The *TERT* amplification status was characterized using fluorescence in situ hybridization. Clinicopathological values and patients’ overall survival were compared between patients with and without TERT amplification. Further sub-cohort analyses were conducted for patients with pT1N0-3 tumor stage. Eighty-One patients (12.6%) exhibited *TERT* amplification. Patients with amplified *TERT* showed significantly worse overall survival (median OS: 22.6 vs. 36.8 months, *p* = 0.009). Interestingly, *TERT* amplification could be characterized as an independent risk factor for worse overall survival in multivariate analysis in patients with pT1N0-3 tumor stage (HR = 2.440, 95% CI 1.095–5.440, *p* = 0.029). In this study, we describe the *TERT* amplification status as an independent risk factor for worse survival in patients diagnosed with esophageal adenocarcinoma at pT1N0-3 tumor stage, encompassing cases involving tumor infiltration of the lamina propria, muscularis mucosae, and/or submucosa. Based on our findings, we put forth the proposition that evaluating the *TERT* amplification status may serve as a valuable tool in identifying a specific subgroup of patients, namely those with *TERT* amplification and pT1N0-3 tumor-stage esophageal adenocarcinoma. The patients of this subgroup could potentially benefit from enhanced follow-up protocols, more aggressive treatment approaches, or possible targeted *TERT* inhibition therapies, all aimed at improving their overall clinical outcomes.

## Introduction

Esophageal cancer is associated with high mortality, and the established treatment strategies, including operative resection, are connected to high morbidity^1^. Only 55.9% of patients, who underwent curatively intended total minimally invasive Ivor-Lewis esophagectomy, show a 5-years overall survival^2^. Recently, classical risk factors, such as histological lymph node metastases or higher T-stage, have been expanded to include the regression grade of lymph node metastases after neoadjuvant treatment. Complete lymph node metastases regression is the most favorable factor for patients’ survival in individuals with esophageal adenocarcinoma. Interestingly, there was a discordance between primary tumor regression and lymph node regression in over 20% of the investigated patients^3^. Despite these promising advances in morphological pathological examination, further biomarkers are required to identify patients with early disease recurrence, particularly in low-risk groups based on established risk classifications.

Hanahan et al. described the hallmarks of cancer over 20 years ago and these have been continuously expanded since then. Evading apoptosis was already outlined in the initial version^4^. Telomere length functions as a mitotic lock under physiological conditions, shortening with each mitosis. When telomere length falls below a critical threshold, cells undergo apoptosis^5^. To circumvent this programmed cell death, cancer cells can restore telomere length by activating telomerase. This enables telomere maintenance and ensures theoretically unlimited mitotic divisions^6^. Telomerase can be upregulated through various mechanisms, including amplification, promotor mutations, or promotor methylation of *TERT* (hTERT), which encodes for the catalytic subunit of telomerase. These upregulations could potentially be associated with changes in patients’ survival across several cancer entities^6,7^. For instance, patients diagnosed with *TERT* promotor mutations in thyroid carcinomas showed not only higher tumor stages or more frequent distant metastases, but also experienced significantly worse survival^8^. These promotor mutations lead to an elevated mRNA expression^9^. *TERT* gene amplification has also been linked to increased TERT mRNA expression and worse overall survival in breast cancer^10^. Furthermore, *TERT* amplification was associated with worse patient survival in acral lentiginous melanoma^11^. Due to these findings, telomerase inhibition is currently under investigation in the cancer treatment^12,13^.

Genetic modifications within *TERT* have shown a potential association with patients’ survival in esophageal cancer. The presence of small nucleotide polymorphisms (SNPs) has been identified as an alteration linked to varying incidences of esophageal cancer (increased or decreased), depending on the specific haplotype^14^. However, the implications for survival stemming from *TERT* amplification in esophageal adenocarcinoma remain unexplored. Therefore, this study seeks to unravel the role of copy number gain of *TERT* in patients with esophageal adenocarcinoma.

## Materials and methods

### Patients and tumor samples

643 patients with esophageal adenocarcinoma, who underwent Ivor-Lewis esophagectomy from 1998 until 2019 at the University Hospital of Cologne with curative intention, were included. The pathological evaluation of the specimen was conducted according to the 7th edition of the Union for International Cancer Control. The tissue microarray (TMA) was constructed with 1,2 mm big tissue cylinders of each tumor, which were punched out with a semi-automated precision instrument. The paraffin-embedded TMA was then cut into 4 µm thick slices for further staining.

### Fluorescence in situ hybridization (FISH) and analysis

Fluorescence in situ hybridization was performed using previously established methods, as outlined in prior publication^15^. Here, we used the marker Zyto Light SPEC TERT/5q31 Dual Color (Zytomed Systems, Germany). Analyses were conducted independently by two experienced pathologists (S. L. and A. Q.) with the immunofluorescent microscope Leica DM5500B (Leica Biosystems, Germany) at 63X. FISH stainings were considered positive (amplified) if the *TERT* signal (green) was on average at least two times higher than the centromere-signal (red) or on average at least 6 gene copies could be counted per cell in 20 analyzed tumor cells. In order to uphold the integrity and credibility of our test results, we have selectively included samples that meet the following criteria: (1) Negative control signals presence within each TMA sample, encompassing elements like blood cells and endothelial vascular cells. (2) Evident and distinct signals in the tumor nuclei.

Furthermore, we conducted a comparative analysis of the value distributions across clinical samples from various years. In this analysis, we did not observe any significant disparities in the distribution patterns.

### Statistical analysis

Follow-up and clinicopathological values were collected prospectively and analyzed retrospectively. Overall survival was considered as the time between surgery and death or loss of follow-up. All analyses were performed with IBM SPSS Statistics (Version 28.0.1.1). Significance was considered if the *p* value was below 0.05. A comparison of qualitative values was conducted with the Chi-Square test. Kaplan–Meier curves and the log-rank test were used for survival analyses. Interdependencies of clinicopathologic and survival data were analyzed with univariate and multivariate Cox regression analyses.

### Ethics approval

This study was performed in line with the principles of the Declaration of Helsinki. Approval was granted by the Ethics Committee of the University Hospital of Cologne (ethics committee number: 21-1146).

### Consent to participate

Informed consent was obtained from all individual participants included in the study.

## Results

This study included 643 patients with esophageal adenocarcinoma, who underwent Ivor-Lewis esophagectomy. Median overall survival of the total study cohort is 33.3 months after surgery. Clinicopathologic values are depicted in Table 1. 65.8% of the included patients (n = 423) received additional neoadjuvant (Radio-)chemotherapy. Pathologically detected lymph node metastases were observed in 368 patients (57.2%). We evaluated the *TERT* amplification status with FISH. 81 patients (12.6%) were evaluated as *TERT*-amplified. The rest of the cohort was labeled as not amplified (87.4%). We built two subgroups according to the amplification status and compared the clinicopathological data of these two groups. In this context, the *TERT* amplified group comprises a significantly higher proportion of male patients (*p* = 0.040). Furthermore, patients displaying a *TERT* amplification were more likely to have lymph node metastases (*p* = 0.018). No significant differences in copy number gain could be detected after neoadjuvant therapy compared to patients undergoing primary surgery (*p* = 0.158).

**Table 1:** General clinicopathological values of the total study population as well as the *TERT* not amplified and amplified group.

Characteristic	Total	TERT not amplified	TERT amplified	*p* value
	n (%)	n (%)	n (%)	
No. of patients	643 (100)	562 (100)	81 (100)	
Sex				**0.040**
Male	567 (88.2)	490 (87.2)	77 (95.1)	
Female	76 (11.8)	72 (12.8)	4 (4.9)	
Age				0.355
< 65	364 (56.6)	322 (57.3)	42 (51.9)	
≥ 65	279 (43.4)	240 (42.7)	39 (48.1)	
Median overall survival (months) (Range)	33.3 (25.5–41.1)	36.8 (27.2–46.4)	22.6 (13.6–31.6)	
Neoadjuvant therapy				0.158
No	219 (34.1)	197 (35.1)	22 (27.2)	
Yes	423 (65.8)	364 (64.8)	59 (72.8)	
Unknown	1 (0.1)	1 (0.1)	0 (0.0)	
(y)pT				0.412
0	4 (0.6)	4 (0.7)	0 (0.0)	
1	130 (20.3)	117 (20.8)	13 (16.0)	
2	101 (15.7)	86 (15.3)	15 (18.5)	
3	395 (61.4)	342 (60.9)	53 (65.4)	
4	13 (2.0)	13 (2.3)	0 (0.0)	
(y)pN				**0.018**
0	275 (42.8)	246 (43.8)	29 (35.8)	
1	183 (28.5)	166 (29.5)	17 (21.0)	
2	88 (13.7)	73 (13.0)	15 (18.5)	
3	97 (15.1)	77 (13.7)	20 (24.7)	
L				0.279
0	290 (45.1)	258 (45.9)	32 (39.5)	
1	353 (54.9)	304 (54.1)	49 (60.5)	
V				0.647
0	469 (72.9)	413 (73.5)	56 (69.1)	
1	70 (10.9)	59 (10.5)	11 (13.6)	
2	104 (16.2)	90 (16.0)	14 (17.3)	
G				0.489
1	2 (0.3)	2 (0.4)	0 (0.0)	
2	122 (19.0)	107 (19.0)	15 (18.5)	
3/4	91 (14.2)	84 (14.9)	7 (8.6)	
Not applicable	423 (65.7)	364 (64.8)	59 (72.8)	
Unknown	5 (0.8)	5 (0.9)	0 (0.0)	

Significant values are in [bold].

*TERT* Telomerase reverse transcriptase.

**Figure 1:**
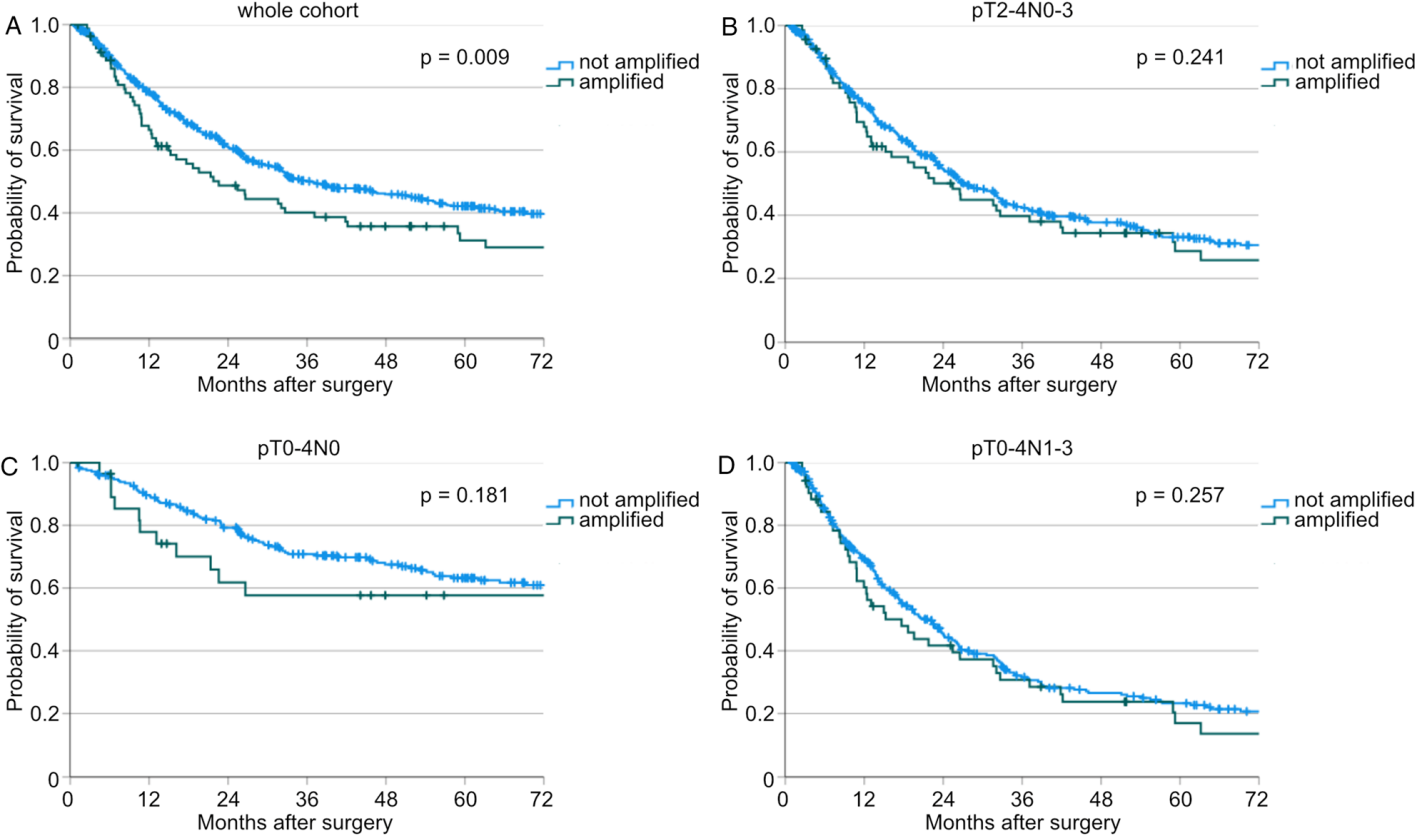
Kaplan-Meier curves for overall survival of patients with not amplified and amplified *TERT* (**A**) of the whole study cohort (n(not amplified) = 562, n(amplified) = 81, *p* = 0.009), (**B**) of patients with pT2-4N0-3 (n(not amplified) = 441, n(amplified) = 68, *p* = 0.241), (**C**) of patients with pT0-4N0 (n(not amplified) = 246, n(amplified) = 29, *p* = 0.181), and (**D**) pT0-4N1-3 (n(not amplified) = 316, n(amplified) = 52, *p* = 0.257).

*TERT* amplification is pathophysiologically correlated with increased cancer cell immortality and tumor growth^6^. Hence, we compared the overall survival of the patient cohort with *TERT* amplification to that of the cohort without *TERT* amplification. Patients with *TERT* amplification showed significantly worse survival compared to the cohort without amplification (median OS: 22.6 vs. 36.8 months, *p* = 0.009, Fig. 1 A). In order to assess the influence of neoadjuvant therapy on this phenomenon, we further subdivided this subgroup into patients who underwent primary surgery (n(amplified) = 22) and those who received additional neoadjuvant therapy (n(amplified) = 59). A similar effect was observed in patients who had undergone neoadjuvant therapy (median OS: 19.6 vs. 30.6 months, *p* = 0.046, Supp. Fig. 1A). Patients with *TERT* amplification, who received primary surgery, showed no significant differences in overall survival compared to patients without amplification (median OS: 26.6 vs. 78.0 months, *p* = 0.197, Supp. Fig. 1B).

To evaluate interdependencies between clinicopathologic values and the overall survival we conducted multivariate Cox regression analyses (Table 2). Here, *TERT* could not be confirmed as an independent factor for worse patients’ survival in the whole patient cohort (HR = 1.226, 95% CI 0.739–2.036, *p* = 0.430). However, higher patients’ age, higher pT-, and higher pN-stage are independent risk factors for worse patients’ survival (age: HR = 1.537, 95% CI 1.039–2.273, p = 0.032, pT: HR = 1.635, 95% CI 1.247–2.143, *p* < 0.001, pN: HR = 1.660, 95% CI 1.372–2.008, *p* < 0.001). Additionally, the survival effect of *TERT* could also not be confirmed in the neoadjuvantly treated whole cohort (HR = 1.039, 95% CI 0.741–1.456, *p* = 0.825).

**Table 2:** Multivariate Cox regression analyses of the total study cohort.

Characteristic	Borders	Hazard Ratio	95% confidence interval	*p* value
Sex	Female vs male	0.645	0.367–1.134	0.127
Age	≥ 65 vs < 65	1.537	1.039–2.273	**0.032**
Neoadjuvant therapy	Yes vs no	1.342	0.835–2.154	0.224
(y)pT	≥ 2 vs < 2	1.635	1.247–2.143	** < 0.001**
(y)pN	≥ 1 vs 0	1.660	1.372–2.008	** < 0.001**
V	≥ 1 vs 0	0.793	0.623–1.010	0.061
G	≥ 1 vs 0	1.208	0.825–1.768	0.331
TERT	Amplified vs not amplified	1.226	0.739–2.036	0.430

Significant values are in [bold].

*TERT*: telomerase reverse transcriptase.

Given that the *TERT* amplification status did not emerge as an independent risk factor for worse overall survival but did demonstrate significantly worse patient survival in the log-rank test, we proceeded to conduct additional sub-analyses. We divided our patient cohort into different cohorts by the TNM stage. Interestingly, no impact of *TERT* amplification on patient survival in patient cohorts with high T-stage (pT2-4N0-3), without lymph node metastases (pT0-4N0), or with lymph node metastases (pT0-4N1-3) could be detected (p(pT2-4N0-3) = 0.241, p(pT0-4N0) = 0.181, p(pT0-4N1-3) = 0.257, Fig. 1B–D). In contrast to these subgroups, we analyzed all patients with a pT1N0-3 tumor stage. 130 patients could be included. Of these patients, *TERT* amplification could be detected in 13 patients (10.0%). All clinicopathologic values are stated in Table 3. In this cohort, older patients were diagnosed more often with a *TERT* amplification (*p* = 0.033).

**Table 3:** General clinicopathological values of the subgroup of all patients with pT1N0-3 tumor stage of EAC as well as the *TERT* not amplified and amplified group.

Characteristic	Total	TERT not amplified	TERT amplified	*p* value
	n (%)	n (%)	n (%)	
No. of patients	130 (100)	117 (100.0)	13 (100.0)	
Sex				0.916
Male	119 (91.5)	107 (91.5)	12 (92.3)	
Female	11 (8.5)	10 (8.5)	1 (7.7)	
Age				**0.033**
< 65	76 (58.5)	72 (61.5)	4 (30.8)	
≥ 65	54 (41.5)	45 (38.5)	9 (69.2)	
Median overall survival (months) (range)	158.4 (95.5–221.3)	181.8 (124.9–238.8)	15.0 (2.8–27.2)	
Neoadjuvant therapy				0.805
No	86 (66.2)	77 (65.8)	9 (69.2)	
Yes	44 (33.8)	40 (34.2)	4 (30.8)	
(y)pT				–
0	0 (0.0)	0 (0.0)	0 (0.0)	
1	130 (100)	117 (100.0)	13 (100.0)	
2	0 (0.0)	0 (0.0)	0 (0.0)	
3	0 (0.0)	0 (0.0)	0 (0.0)	
4	0 (0.0)	0 (0.0)	0 (0.0)	
(y)pN				0.656
0	103 (79.2)	94 (80.3)	9 (69.2)	
1	22 (16.9)	19 (16.2)	3 (23.1)	
2	4 (3.1)	3 (2.6)	1 (7.7)	
3	1 (0.8)	1 (0.9)	0 (0.0)	
L				0.180
0	91 (70.0)	84 (71.8)	7 (53.8)	
1	39 (30.0)	33 (28.2)	6 (46.2)	
V				0.568
0	103 (79.2)	94 (80.3)	9 (69.2)	
1	1 (0.8)	1 (0.9)	0 (0.0)	
2	26.20.0)	22 (18.8)	4 (30.8)	
G				0.796
1	1 (0.8)	1 (0.9)	0 (0.0)	
2	68 (52.3)	60 (51.3)	8 (61.5)	
3/4	15 (11.5)	14 (12.0)	1 (7.7)	
Not applicable/unknown	46 (35.4)	42 (35.8)	4 (30.8)	
TERT				–
Not amplified	117 (90.0)	117 (100.0)	0 (0.0)	
Amplified	13 (10.0)	0 (0.0)	13 (100.0)	

Significant values are in [bold].

*TERT*: telomerase reverse transcriptase.

**Figure 2:**
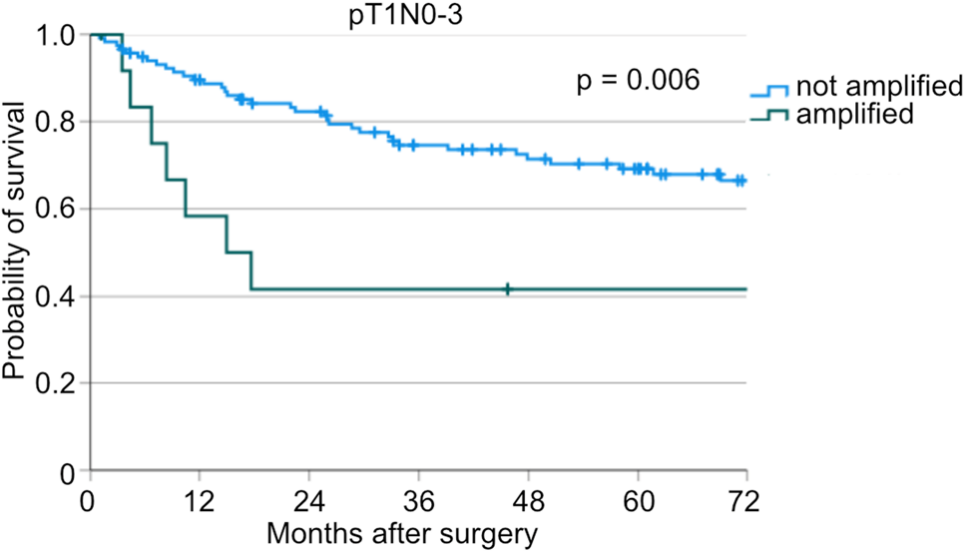
Kaplan-Meier curve for overall survival of patients with not amplified and amplified *TERT* of all patients with pT1N0-3 (n(not amplified) = 117, n(amplified) = 13, *p* = 0.006).

Survival analyses show significantly worse patients’ survival in the subgroup of patients with (y)pT1N0-3-stage (median OS: 15.0 vs. 181.8 months, *p* = 0.006, Fig. 2). Further analyses in this subcohort regarding neoadjuvant treatment revealed, that *TERT* amplification is correlated with worse survival in patients, who were primarily operated, but not in patients after neoadjuvant therapy (p = 0.014 vs. *p* = 0.101, Supp. Fig. 1C, D).

Multivariate Cox regression analyses could verify the *TERT* amplification status in patients with a pT1N0-3 tumor stage as an independent risk factor for poor survival (HR = 2.440, 95% CI 1.095–5.440, *p* = 0.029, Table 4). Furthermore, high age, neoadjuvant therapy, and a high pN-stage showed to be independent risk factors for worse patients’ survival (age: HR = 2.067, 95% CI 1.130–3.780, *p* = 0.018, neoadjuvant therapy: HR = 2.196, 95% CI 1.189–4.055, *p* = 0.012, pN: HR = 2.112, 95% CI 1.404–3.176, *p* < 0.001). The *TERT* amplification status did not prove to be an independent risk factor for worse survival in the subgroup analysis of primarily operated patients of the pT1N0-3-cohort (HR = 2.241, 95% CI 0.703–7.139, *p* = 0.172).

**Table 4:** Multivariate Cox regression analyses of patients with pT1N0-3 tumor stage.

Characteristic	Borders	Hazard Ratio	95% confidence interval	*p* value
Age	≥ 65 vs < 65	2.067	1.130–3.780	**0.018**
Neoadjuvant therapy	Yes vs no	2.196	1.189–4.055	**0.012**
(y)pN	≥ 1 vs 0	2.112	1.404–3.176	** < 0.001**
V	≥ 1 vs 0	0.809	0.535–1.223	0.316
TERT	Amplified vs not amplified	2.440	1.095–5.440	**0.029**

Significant values are in [bold].

*TERT*: telomerase reverse transcriptase.

In summary, we can characterize the *TERT* amplification status as an indicator of poorer patient survival. Though not in the total patient cohort, this characterization could be substantiated as an independent risk factor in patients with a pT1N0-3 tumor stage.

## Discussion

We present a retrospective, single-center cohort study that includes 643 patients with operable esophageal adenocarcinoma. In this study, we have demonstrated that patients with *TERT* amplification experience poorer overall survival. Additionally, a copy number gain could be predominantly observed in male patients. This phenomenon has been previously observed in *TERT*-amplified tumors across various other entities, such as acral melanoma, breast cancer, and lung neuroendocrine tumors^10,16,17^. Interestingly, patients with ulceration or higher tumor thickness showed significantly higher *TERT* copy numbers in patients with acral melanoma^16^. Furthermore, *TERT* copy number alterations in solitary fibrous tumors and *TERT* mutations in non-small cell lung cancer could be associated with a higher risk of metastasis^18,19^. Interestingly, RNA sequencing analysis unveiled a connection between the expression of *TERT* and genes related to cell migration. This observation implies that TERT’s function extends beyond conferring immortality to cancer cells; it also appears to facilitate cell migration and contribute to metastatic processes^20^.

65.8% of the included patients received neoadjuvant therapy. To address the influence of neoadjuvant therapy on the *TERT* amplification status and its value as a biomarker for worse patients’ survival, we conducted additional subanalyses of patients, who received neoadjuvant therapy or surgery solely. In this context, *TERT* amplification was notably associated with a significant decline in overall survival in patients undergoing neoadjuvant therapy (median OS: 19.6 vs. 30.6 months, *p* = 0.046). On the contrary, this effect could not be confirmed in patients after primary surgery (*p* = 0.197). To assess these differences, future prospective studies are needed.

In our study, the impact of the *TERT*-amplification status could not be substantiated in multivariate Cox regression analyses within the whole patient cohort (*p* = 0.430). Nevertheless, we established tumor sub-cohorts for more in-depth examination. Importantly, the *TERT* amplification status was revealed as an indicator of compromised overall survival among patients with a pT1N0-3 tumor stage (median OS: 15.0 vs. 181.8 months, *p* = 0.006). Additionally, *TERT* copy number gain proved to be an independent risk factor for worse patient survival in this sub-group (HR = 2.440, 95% CI 1.095–5.440, *p* = 0.029). *TERT* primarily exhibits activity in malignant processes in adults. Studies involving premalignant processes have revealed heightened TERT expression in these precursor lesions, such as adenomas in the colon and ductal breast carcinoma in situ^21^. However, it’s important to note that these findings may not be applicable to every patient with esophageal adenocarcinoma, as substantial variations in the prevalence of *TERT* mutations based on race and sex have been previously reported depending on the cancer entity^22,23^. Our study cohort predominantly consists of Caucasian patients. However, it is important to note that our data bank does not provide information about patients’ racial background. Further prospective studies must be performed to verify the impact of *TERT* amplification in defined patient subgroups.

Counterintuitively, neoadjuvant therapy was described as a factor for worse patient survival in our pT1N0-3 tumor stage subcohort. However, this observation could be rationalized by the small sample size and by the fact that patients with more advanced tumor stages often receive neoadjuvant therapy in alignment with international guidelines, which improves individual patient survival. Nevertheless, the survival outcomes of these patients remain inferior compared with patients with earlier tumor stages.

Patients with early tumor stage and absence of pathological lymph nodes during the initial staging meet the criteria outlined by international guidelines for consideration of exclusive endoscopic resection^24^. All patients included in this study received Ivor-Lewis esophagectomy. Since *TERT*-amplification showed to be an independent risk factor for worse patients´ survival in our subcohort of patients with pT1N0-3 tumor stage, future studies to evaluate the prognostic value of *TERT* in patients undergoing exclusively endoscopic treatment could be of great clinical value. We hypothesize, that *TERT*-amplified patients should receive surgical resection despite the early pT tumor stage.

*TERT* could serve not only as a prognostic biomarker but also as a potential therapeutic target. Mouse experiments have demonstrated that cancer cells exhibiting *TERT* copy number gain are responsive to telomerase inhibitors in cases such as melanoma^13^. The thio-phosphoramidate oligonucleotide inhibitor of telomerase, imetelstat, was evaluated in-vitro for the treatment of esophageal squamous cell cancer cells. Promisingly, imetelstat could inhibit telomerase, which resulted in a decreased colony formation ability and decreased cancer cell growth. Furthermore, increased DNA double-strand breaks could be detected. This effect could be intensified when combining radiotherapy and imetelstat. This could qualify imetelstat as a possible therapy agent in a multimodal therapy regime^12^.

Taken together, we could describe the *TERT* amplification status as an independent risk factor associated with poorer patient survival. *TERT* amplification status identifies a high-risk patient group in a generally low-risk patient cohort, based on clinically established clinicopathological values.

## Conclusions

This study aims to shed light on the relationship between *TERT* amplification status and patient survival in individuals diagnosed with esophageal adenocarcinoma. Our data demonstrates that *TERT* amplification status is a significant risk factor associated with reduced overall survival in a specific patient subgroup presenting with a pT1N0-3 tumor stage. These findings underscore the potential value of *TERT* amplification as a predictive marker for identifying higher-risk patients who may in the future benefit from more intensive follow-up protocols. Moreover, as additional prospective studies surface, it may warrant consideration of adjuvant therapies for this patient cohort, despite their early pT tumor stage.

### Supplementary Information

Supplementary Figure 1.

## Data Availability

The datasets generated and analyzed during the current study are available from the corresponding author on reasonable request.
