# Supplementary material for: Copy-number-gain of telomerase reverse transcriptase (hTERT) is associated with an unfavorable prognosis in esophageal adenocarcinoma
Source: Sci Rep. 2023 Oct 17;13:17699. doi: 10.1038/s41598-023-44844-7 (PMC10582081; doi:10.1038/s41598-023-44844-7)

**Copy-number-gain of telomerase reverse transcriptase (hTERT) is associated with an unfavorable prognosis in esophageal adenocarcinoma**

Su Ir Lyu^1^, Felix C. Popp^2^, Adrian Georg Simon^1^, Anne Maria Schultheis^1^, Thomas Zander^3^, Caroline Fretter^1^, Wolfgang Schröder^2^, Christiane J. Bruns^2^, Thomas Schmidt^2^, Alexander Quaas^1^, Karl Knipper^2*^

**Supplement Material**

**Supp. Figure 1**

Kaplan-Meier curves for overall survival of patients with not amplified and amplified TERT of (A) all patients of the whole cohort after neoadjuvant therapy (n(not amplified) = 364, n(amplified) = 59, p = 0.046), (B) all patients of the whole cohort after primary surgery (n(not amplified) = 197, n(amplified) = 22, p = 0.197) (C) all patients of the pT1N0-3 cohort after neoadjuvant therapy (n(not amplified) = 40, n(amplified) = 4, p = 0.101), and (D) all patients of the pT1N0-3 cohort after primary surgery (n(not amplified) = 77, n(amplified) = 9, p = 0.014).


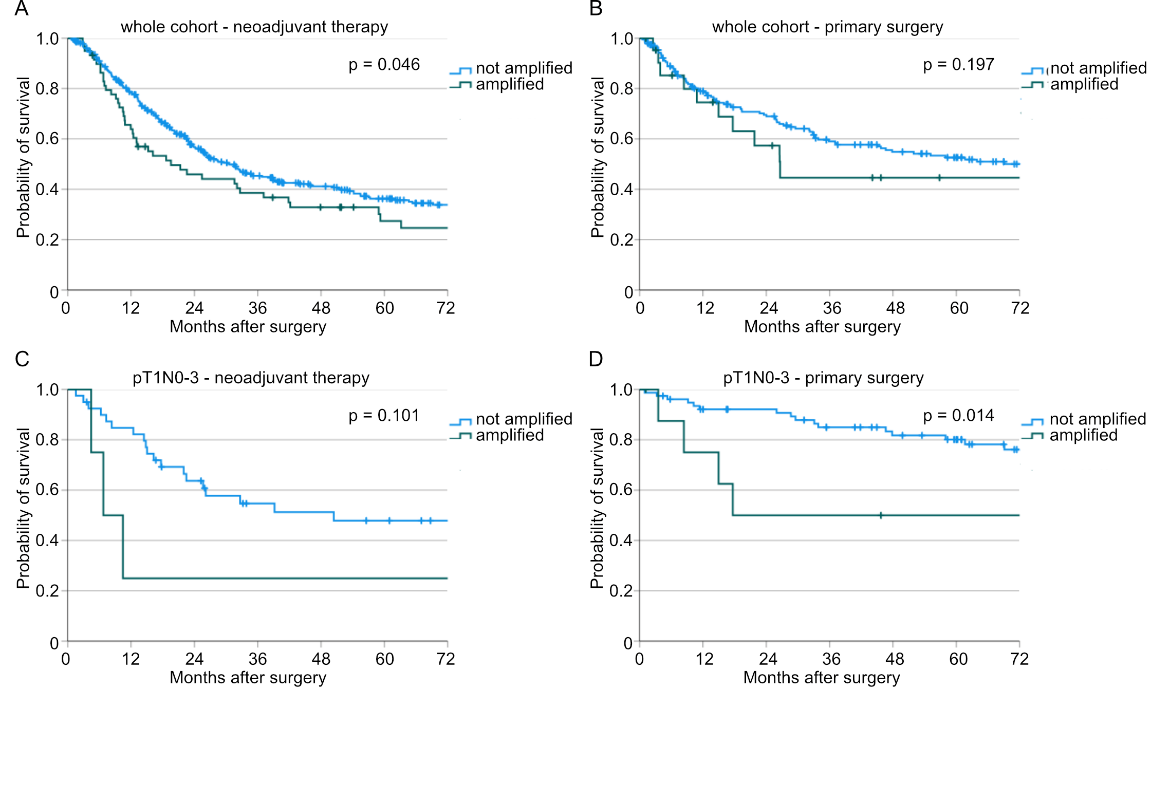

Supplement: Supplementary file 1 — Supplementary Figure 1. [file 41598_2023_44844_MOESM1_ESM.docx]
